# Two-Dimensional Black Phosphorus: An Emerging Anode Material for Lithium-Ion Batteries

**DOI:** 10.1007/s40820-020-00453-x

**Published:** 2020-06-05

**Authors:** JiPing Zhu, GuangShun Xiao, XiuXiu Zuo

**Affiliations:** School of Materials Science and Engineering, Hefei University of Technology, Hefei, 230009 People’s Republic of China

**Keywords:** Two-dimensional material, Black phosphorus, Lithium-ion batteries

## Abstract

Two-dimensional black phosphorus (2D BP), an emerging material, has aroused tremendous interest once discovered. This is due to the fact that it integrates unprecedented properties of other 2D materials, such as tunable bandgap structures, outstanding electrochemical properties, anisotropic mechanical, thermodynamic, and photoelectric properties, making it of great research value in many fields. The emergence of 2D BP has greatly promoted the development of electrochemical energy storage devices, especially lithium-ion batteries. However, in the application of 2D BP, there are still some problems to be solved urgently, such as the difficulty in the synthesis of large-scale high-quality phosphorene, poor environmental stability, and the volume expansion as electrode materials. Herein, according to the latest research progress of 2D BP in the field of energy storage, we systematically summarize and compare the preparation methods of phosphorene and discuss the basic structure and properties of BP, especially the environmental instability and passivation techniques. In particular, the practical application and challenges of 2D BP as anode material for lithium-ion batteries are analyzed in detail. Finally, some personal perspectives on the future development and challenges of BP are presented.

## Highlights

- The preparation methods, basic structure, and properties as well as environmental instability and passivation techniques of two-dimensional black phosphorus are systematically summarized and analyzed.
- The application of anode materials based on two-dimensional black phosphorus in lithium-ion batteries in recent years is wholly reviewed.

## Graphical abstract

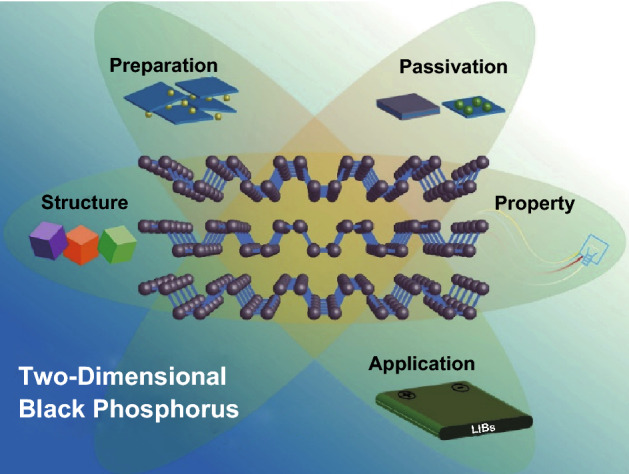

## Introduction

The development of materials promotes the progress of human society, and the discovery of each new material will cause revolutionary changes in science and technology. In 2004, Novoselov et al. [1] successfully exfoliated graphene from bulk graphite via the mechanical cleavage method. The emergence of this new material broke people’s understanding of traditional materials and opened up a new field of two-dimensional (2D) materials. Graphene is called 2D material because it consists of just one layer of *sp*^2^-bonded carbon atoms forming hexagonal honeycomb lattices. The special 2D structure provides graphene with a series of unexpected properties, such as large special surface [2], strong mechanical properties [3], and excellent electronic, optical and thermal properties [4–6]. These incredible properties have been widely applied in energy storage devices, biomedical engineering, optics, and thermally conductivity [7–10]. The success of graphene has aroused intensive interest in 2D materials.

In general, materials where electrons can move freely on three dimensions of non-nanoscale are called 3D materials, while materials where electrons can move freely on only two dimensions of nanoscale are called 2D materials [11]. The rise of graphene has driven the rapid development of 2D material family, such as transition metal dichalcogenides (TMDS) [12, 13], hexagonal boron nitride (h-BN) [14, 15], transition-metal carbides and nitrides (MXenes) [16] and 2D black phosphorus (BP) [17]. Most of these 2D materials have the characteristics of ordered structure, two-dimensional plane growth, and three-dimensional ultra-thin. Nevertheless, there are no strict rules on the thickness of 2D materials, which can be single layer or multilayers. The individual layers are attracted by weak van der Waals forces, which makes it possible to regulate the number of layers of 2D materials [18, 19]. By adjusting the number of layers, the energy band and electrical characteristics can be controlled, so that 2D materials can behave as conductors, semiconductors, and insulators [20]. In addition, atoms or molecules in a single plane are connected by strong covalent or ionic bonds and are completely exposed, making 2D materials with high strength, high transparency, high-speed electron transmission, and modifiable characteristics [21]. These fascinating characteristics lead us to believe that 2D materials are bound to achieve disruptive innovations in semiconductors, sensors, energy storage devices, flexible electronics, photocatalyst, and other fields [20, 22–24]. At present, the USA, the UK, South Korea, Japan, Singapore, and other countries have promoted the research of 2D materials to the level of national strategy. However, the study of 2D materials has just begun.

In the process of studying 2D materials, people have some new understanding of them. For example, it has been proven that graphene has excellent performance in many aspects, but with the expansion of the application field, the zero bandgap of graphene has gradually become its fatal disadvantage. Furthermore, most MXenes also show limited energy bands [25]. The limited bandgap makes them more metal-like, making it difficult to meet the requirements of semiconductor and optoelectronic applications [26]. In addition, the wide bandgap of h-BN endows it with the characteristics of insulator, which also restricts its utilization field [27]. While TMDs have low carrier mobility, which affects the performance of the device in practical application [28]. Therefore, people continue to advance on the road of exploring a 2D material with well-balanced performance.

Until 2014, the emergence of 2D BP pointed out a new direction for the study of 2D materials. According to the theoretical research, 2D BP is regarded as an advanced material that integrates unprecedented properties of graphene, TMDs and other 2D materials [29]. The 2D BP breaks the bound energy band property of graphene, MXenes and h-BN, and presents a tunable energy band within the range of 0.3–2 eV. It also shows that the carrier mobility of 1000 cm^2^ V^−1^ s^−1^ is much higher than that of TMDs [30]. These characteristics make it a natural semiconductor that has had a huge impact on the fields of photonics, electronics, and sensors [31–33]. Additionally, 2D BP also exhibits excellent mechanical, electrochemical, and thermodynamic properties, showing great research value in ultra-light materials, energy storage devices, flexible electronics, etc. [34, 35]. In fact, as early as 1914, when Bridgman attempted to convert white phosphorus (WP) into red phosphorus (RP) under high pressure and high temperature, the 3D bulk BP was accidentally synthesized [36]. This makes black phosphorus turn into the most stable allotrope of phosphorus in the thermodynamic properties at ambient conditions. Nevertheless, it was not until 2014 that the 2D BP was prepared. Using scotch tape to strip the BP flake repeatedly to destroy the weak van der Waals force between layers, the monolayer or few layers of BP with 2D structure (the following “BP” terms refer to 2D structural BP) was obtained. These thin-layer BP with atomic thickness are also known as “phosphorene” [37]. This also promotes the development of 0D BP quantum dots (BPQDs) [38]. BPQDs have unique optical properties and good biocompatibility, which makes it have unparalleled advantages in biomedical fields such as cancer treatment, drug delivery, and cellular tracking [39–41]. There is no doubt that this kind of omnipotent 2D material with well-balanced properties has shown great research value and has aroused tremendous interest of researchers in many fields.

The emergence of 2D BP also promoted the development of new energy field. With the progress of society and technology, the existing energy materials are far from meeting people’s needs, which spurrings people’s continuous exploration in the field of new energy. The electrochemical energy storage devices, such as lithium-ion batteries (LIBs), sodium-ion batteries (SIBs), lithium-sulfur batteries (LSBs), magnesium-ion batteries (MIBs), and supercapacitors are developing rapidly. Among these energy storage devices, LIBs have been widely applied in 3C market (mobile phone, computer, camera, etc.) with its the most mature system. However, the application scope of LIBs is mostly limited to these small-devices and has limited applications in the power battery market. For this reason, researchers have been committed to exploring high-performance LIBs with high capacity, high rate, and long life. The electrode material determines the overall performance of LIBs. Therefore, BP with high theoretical specific capacity of 2596 mAh g^−1^ undoubtedly provides a new opportunity for the further development of LIBs [35]. In recent years, a large number of theoretical and experimental studies have been carried out on BP and BP-based electrode materials in order to seek new breakthrough in the field of LIBs.

Consequently, a detailed review of BP and BP-based anode materials for LIBs are presented in this paper, aiming to provide reference for the research of this emerging material in the field of new energy. In the following context, firstly, the different preparation methods of phosphorene are summarized and compared. Secondly, the fundamental structure and properties of BP are introduced. Thirdly, the environmental instability and passivation techniques of thin-layer BP are discussed in detail. Then we focus on the latest research progress of BP-based anode materials in LIBs. Finally, our insight on the opportunities and challenges of BP in this field are put forward.

## Preparation of Phosphorene

Since phosphorene (monolayer or few-layer BP) was originally prepared by an inefficient scotch tape mechanical cleavage method, a large number of experiments have been carried out to explore a more reliable method for the fabrication of high-quality phosphorene. In recent years, several preparation methods have been developed for fundamental research. As a general rule, methods can be divided into “top-down methods” (such as mechanical cleavage) and “bottom-up methods” (such as CVD).

### Top-Down Methods

Since bulk BP was discovered as early as 100 years ago, researchers initially tended to obtain phosphorene by peeling directly from the bulk BP. Such methods as breaking the weak van der Waals force between layers by physical or chemical means to obtain phosphorene are known as the top-down methods.

#### Mechanical Exfoliation

**Fig. 1:**
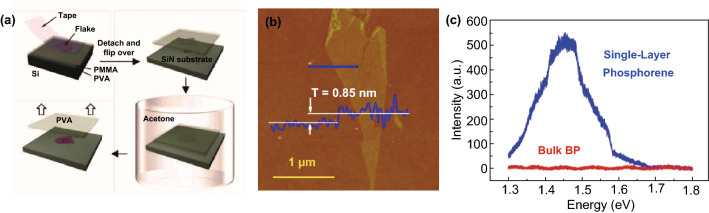
**a** The process of preparing phosphorene by mechanical exfoliation method [42]. **b** AFM image and **c** PL spectra of monolayer phosphorene prepared by mechanical exfoliation method [44]. Adapted from Refs. [42, 44] with permission

The traditional mechanical exfoliation method is to peel BP repeatedly with scotch tape until the few-layer BP flake is obtained [1]. Then the BP flake are transferred onto Si/SiO_2_ substrate to remove the scotch tape residue with acetone, methanol and isopropyl alcohol, a process similar to Fig. 1a [42]. Finally, the solvent residue is removed under 180 °C conditions to obtain the final product [37, 43, 44]. Liu et al. [44] prepared phosphorene with different layers by this method. From the AFM image in Fig. 1b, it can be observed that the thickness of the monolayer phosphorene is 0.85 nm, which is larger than the theoretical calculation of 0.6 nm. The photoluminescence (PL) spectra (Fig. 1c) show that the energy gap of monolayer phosphorene is 1.45 eV. In addition, the prepared 4–6 nm BP flakes exhibited a hole carrier mobility of 286 cm^2^ V^−1^ s^−1^. Guan et al. [45] improved the traditional scotch tape mechanical exfoliation method. A layer of gold or silver of about 10 nm was deposited on the Si/SiO_2_ substrate to enhance the adhesive force between the bulk BP and the substrate, and then peeled off. The phosphorene prepared by this metal-assisted mechanical exfoliation method has a thickness of ~ 4 nm, a lateral size of ~ 50 µm and a hole carrier mobility of 68.6 cm^2^ V^−1^ s^−1^. It can be seen that the phosphorene prepared by the mechanical exfoliation method has irregular size, uncontrollable layer number, general electrical performance and low efficiency, which can only be used for fundamental structure and property research [46–49].

#### Liquid-Phase Exfoliation

**Fig. 2:**
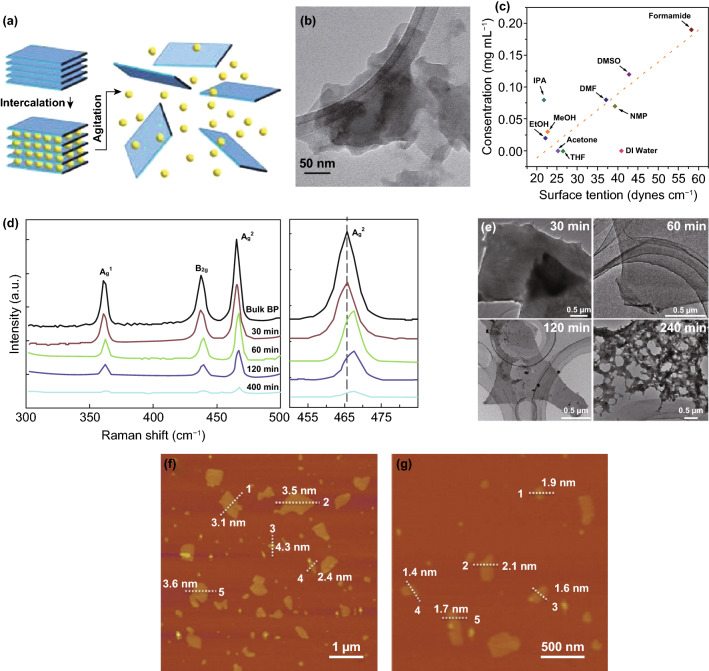
**a** The liquid-phase exfoliation processes of BP [50]. **b** Low TEM image of few-layer BP [51]. **c** Plot of surface tension versus phosphorene concentration in different solvents [57]. **d** Raman spectrum and **e** TEM image of phosphorene under different ultrasonic time [56]. AFM image of phosphorene collected at **f** 2000 rpm and **g** 4000 rpm [57]. Adapted from Refs. [50, 51, 56, 57] with permission

The liquid-phase exfoliation method of BP usually involves three steps: (1) dispersion of the bulk BP in the solvent, (2) exfoliation via ultrasonication, and (3) purification. The detailed process (Fig. 2a) shows that the intercalation of the solvent molecule destroys the weak van der Waals bonds between BP layers, and then the phosphorene can be obtained by ultrasonic dispersion [50].

The choice of solvent plays an important role in liquid-phase exfoliation process. In 2014, Brent et al. [51] used *N*-methyl-2-pyrrolidone (NMP) as solvent to prepare one-to-five layer BP with significant lateral dimensions of 100 nm (Fig. 2b) by liquid-phase exfoliation method for the first time. In general, the selected solvent should have similar surface energy to BP for successful and effcient exfoliation. NMP [31, 52] has the surface energy of 40 mJ m^−2^ that is close to 35–40 mJ m^−2^ of BP. Furthermore, there are some other solvents that can also be used to peel bulk BP, such as dimethylformamide (DMF) [53], *N*-cyclohexyl-2-pyrrolidone (CHP) [54], acetone [55, 56], dimethyl sulfoxide (DMSO) [53] and so on. Zhang et al. [57] explored ten different solvents (Fig. 2c) for liquid-phase exfoliation of BP, and found that using the formamide showed a maximum yield of 38% (with a high concentration of 0.19 mg mL^−1^). Del Rio Castillo et al. [58] also explored the exfoliation effects of different solvents, and the results indicated that the acetone can provide a high exfoliation concentration and the optimum size (thickness and average lateral size of ∼ 7 and ∼ 30 nm).

In order to fully tap the potential of BP, especially in electronic device, it is necessary to prepare large-scale high-quality phosphorene. Therefore, researchers have been exploring the preparation methods and conditions for large-scale BP. In the process of liquid-phase exfoliation, the ultrasonic time has a great influence on the size of phosphorene. Yan et al. [56] used Raman spectrum and transmission electron microscope (TEM) to characterize phosphorene prepared by liquid-phase exfoliation at different ultrasonic time (30, 60, 120, and 480 min). The Raman spectrum (as shown in Fig. 2d) shows that the Raman standard peaks position of BP begins to deviate as the ultrasonic time increases to 60 min. Moreover, it can be seen from the TEM (Fig. 2e) image that the few-layer BP with ultrasonic time of 60 min displays the optimal thickness (average thickness of ~ 2 nm) and horizontal size (lateral dimension up to 10 μm). In addition, during the purification process, Zhang et al. [57] also found that the thickness and horizontal size of phosphorene could be easily controlled by changing the centrifugation speed. The AFM image (Fig. 2f) shows that the average thickness of phosphorene prepared at the centrifugation speed of 2000 revolutions per minute (rpm) is about 3.1–4.3 nm which is equivalent to about 6–8 layers of monolayer BP, and with a lateral size ranging from 50 nm to 1 µm. As the centrifugation speed increases to 4000 rpm, the AFM image (Fig. 2g) indicate that thinner (1.4–2.1 nm, corresponding to 2–4 layers) and smaller (50–300 nm in lateral size) phosphorene can be obtained.

Although the size of the phosphorene can be controlled by changing the ultrasonic time and the centrifugation speed, it is undeniable that this top-down liquid-phase exfoliation method has certain limitations, which often results in non-uniform size of the prepared phosphorene. Besides that, although the yield and efficiency of the liquid-phase exfoliation method are improved compared to the mechanical exfoliation method, they are still relatively low. Accordingly, in order to achieve high-yield and high-efficiency preparation of large-scale uniform phosphorene, some liquid-phase assisted methods have been developed, such as additive-assisted method [32, 59], microwave-assisted method [60], and solvothermal-assisted method [56].

Jing et al. [59] took phytic acid as the surfactant and peeled the uniform large rectangular sheets with length of ~ 24–28 μm, width of ~ 4–6 μm, and height of ~ 3–4 nm (6–8 layers) in DMF by liquid-phase exfoliation. Meanwhile, the BP nanosheets showed a band gap of 1.70 eV. The addition of surfactant can make the interlayer van der Waals bonds more easily broken during the ultrasonic process, thereby enhancing the strip ratio and reducing the damage of ultrasound to the BP structure, so that the prepared phosphorene can maintain a good morphology and thus exhibiting the best property. Bat-Erdene et al. [60] reported an extremely efficient microwave-assisted liquid-phase exfoliation process that can be completed in 12 min, while traditional ultrasonication usually takes several hours [31, 54]. And the prepared phosphorene has the thickness of 4–11 layers and the lateral dimensions of hundreds of nanometers to ~ 4 µm. In recent studies, the liquid-phase exfoliation method has been widely used in the preparation of phosphorene and devices application research. However, as the liquid-phase exfoliation is mostly carried out in organic solvents, it will produce harmful pollutants to the environment. And the organic solvent molecules are easy to adsorb on the surface of phosphorene, affecting its intrinsic properties [61]. Furthermore, the systematic control conditions for the thickness and lateral dimension of phosphorene still need more research.

#### Electrochemical Exfoliation

The electrochemical exfoliation method has the characteristics of simple, ultrafast, and environmental friendly, which has been widely reported as a method for preparing high-quality graphene [62, 63]. This inspired researchers to use electrochemical exfoliation method to prepare phosphorene.

**Fig. 3:**
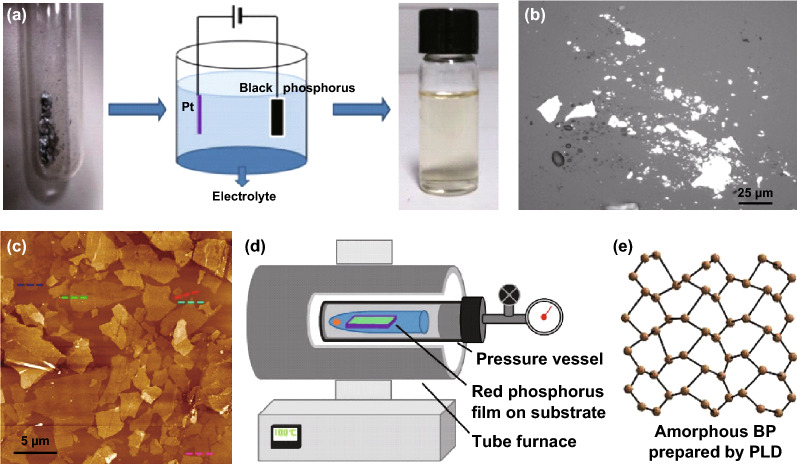
**a** Experimental device of BP electrochemical exfoliation [67]. **b** Optical images of BP nanosheets prepared by electrochemical exfoliation [64]. **c** AFM image of few-layer prepared by electrochemical exfoliation [65]. **d** The device for synthesizing BP thin films by CVD method [72]. **e** The top view of disordered atomic structures of BP [74]. Adapted from Refs. [64, 65, 67, 72, 74] with permission

In the electrochemical exfoliation process (Fig. 3a), the bulk BP as a working electrode, by applying voltage to generate current, the bulk BP can be stripped into phosphorene due to the combined effect of current and electrolyte on the layered structure. The selection of voltage and electrolyte determines whether bulk BP can be successfully detached. Erande et al. [64] used the bulk BP crystal as anode, the platinum wire as counter electrode and 0.05 M Na_2_SO_4_ as electrolyte. The voltage applied to the two-electrode system was +7 V which corresponding to the current of ∼ 1 mA. After ~ 25 min of reaction, the solution turned light yellow, and power off after 90 min. Finally, the phosphorene with atomically thin-layer was obtained by centrifugation. The yield can reach more than 80%, which is higher than the liquid-phase exfoliation method reported in recent years. However, as shown in Fig. 3b, the exfoliated BP nanosheets exhibit a wide range of thickness (1.4–10 nm, corresponding to 3–15 layers) and lateral dimensions (0.5–30 µm). This non-uniform size makes it only have a mobility of 7.3 cm^2^ V^−1^ s^−1^. Li et al. [65] adopted different electrolyte (0.001 M Tetraalkylammonium (TAA) salts and DMSO) and voltage (− 5 V low voltage) to realize rapid expansion and peeling of bulk BP within a few minutes. The yield also exceeds 80%. And the fabricated few-layer BP possess a good uniform size (as shown in Fig. 3c) and electrical property, with the average thickness of ∼ 5 layers, the average lateral area of ∼ 10 μm^2^ and hole carrier mobility of 100 cm^2^ V^−1^ s^−1^. In addition, it is reported that H_2_SO_4_ [66] and Tetrabutylammonium hexafluorophosphate (TBA) [67] can also be used as electrolyte for BP electrochemical exfoliation.

Obviously, high-quality uniform phosphorene can be prepared by choosing the appropriate electrolyte and voltage. The electrochemical exfoliation method opens up new possibilities for the industrial production of phosphorene.

### Bottom-up Methods

It is undeniable that even if the preparation conditions of top-down methods are precisely controlled, the phosphorene prepared still have the characteristics of small size and non-uniformity, and few high-quality phosphorene can be extracted for device. For this reason, researchers are exploring the bottom-up methods to directly synthetize large-scale and high-quality phosphorene.

The chemical vapor deposition (CVD) approach is the most typical bottom-up preparation method, which synthesizes the desired materials by depositing gaseous materials into solid ones [68]. The CVD method has a broad application in the preparation of 2D materials (graphene, TMDS, h-BN, etc.) [12, 15, 66, 69–71], and this method is regard as one of the most prospective bottom-up methods. However, there are few reports on the preparation of phosphorene by CVD due to the limitation of cost, technology and synthesis conditions. Smith et al. [72] reported an in situ CVD type approach in which RP film was directly converted into BP film on the silicon substrate (Fig. 3d). The synthetized BP film possess a large size with an average area of > 3 μm^2^ and a film thickness as thin as four layers. Furthermore, it is believed that a larger size of BP film could be obtained by further optimizing the preparation conditions. Jiang et al. [73] also used CVD approach to grow BP directly on carbon paper to synthetize BP/C composites as the anode materials of LIBs.

Zhang et al. [74] proposed another bottom-up method called pulsed laser deposition (PLD) approach that successfully deposited ultrathin BP film at 150 °C. The prepared BP layer show tunable direct energy gap which decreases from 0.80 to 0.21–0.26 eV as the thickness of BP film increases from 2 to 8 nm. However, the BP film exhibit short-range ordered and distorted lattice (Fig. 3e), which results in a mobility of only 14 cm^2^ V^−1^ s^−1^. Anyway, this PLD technique at relatively low processing temperature provides a new idea for us to obtain ultrathin phosphorene.

Xu et al. [75] reported a gas-phase growth strategy on the insulating silicon substrates, which successfully achieved large-scale and high-quality phosphorene preparation through epitaxial nucleation design and further lateral growth control. The lateral size of the prepared BP films can reach up to millimeters, and the thickness can be adjusted from a few to hundreds of nanometers. Furthermore, the mobility of the BP film is up to 1200 cm^2^ V^−1^ s^−1^, which is much higher than that of phosphorene prepared by top-down methods.

Table 1 summarizes the current preparation methods of phosphorene and analyzes the characteristics of each method. Mechanical exfoliation method is only used in the early exploration of the basic structure and properties of BP. Liquid-phase exfoliation method has become the main method to prepare phosphorene because of its simplicity and low cost, but the yield and size control still need to be further improved. Electrochemical exfoliation shows high stripping efficiency and yield, which has great application potential in the industrial production of phosphorene. The direct bottom-up chemical synthesis methods possess broad application prospects in the preparation of controllable large-scale and high-quality phosphorene, but the technology is still immature which need further exploration in the preparation conditions, size and cost control.

**Table 1:** The main preparation methods and comparison of phosphorene at present

Method		Experiment	Thickness	Carrier mobility (cm^2^ V^−1^ s^−1^)	Characteristic	References
Top-down	Mechanical exfoliation	Scotch tape-based mechanical exfoliation	4 ~ 6 nm	286	Long time consuming, low yield, uncontrolled size	[44]
		metal-assisted mechanical exfoliation	~4 nm	68.6	long time consuming, low yield, uncontrolled size, ~ 50 µm lateral dimension	[45]
	Liquid-phase exfoliation	Sonic exfoliated in NMP for 24 h	3–5 layers	–	200 × 200 nm^2^ lateral dimension	[51]
		Sonic exfoliated in formamide, and centrifuged at 9000 rpm	3 ± 1 layers	–	50–300 nm lateral dimension, 38% yield	[57]
		Phytic acid-assisted exfoliation in DMF	6–8 layers	–	Several tens of micrometers lateral dimension	[59]
		Microwave-assisted exfoliation in NMP	4–11 layers	–	Hundreds of nanometers up to ≈ 4 µm lateral dimension, < 12 min processing time	[60]
	Electrochemical exfoliation	Two-electrode system (Pt and bulk BP), electrolyte (0.05 M Na_2_SO_4_), +7 V voltage	3–15 layers	7.3	0.5 to 30 µm lateral dimension, yield excess 80%, non-uniform size	[64]
		Two-electrode system (Pt and bulk BP), electrolyte (0.001 M TAA in DMSO), − 5 V voltage	~5 layers	100	∼10 μm^2^ lateral dimension, several minutes processing time, > 80% yield	[65]
Bottom-up	CVD	Conversion of RP film to BP film by CVD	~4 layers	–	Controllable size, a variety of lateral size samples	[72]
	PLD	Deposited BP film at 150 °C by PLD	2–8 nm	14	Low processing temperature, tunable direct band gap	[74]
	Gas-phase growth strategy	BP is grown directly on the insulating silicon substrates	A few to hundreds of nanometers	1200	Controllable thickness and lateral dimension can up to millimeters	[75]

## Structure and Properties of BP

### Structure

**Fig. 4:**
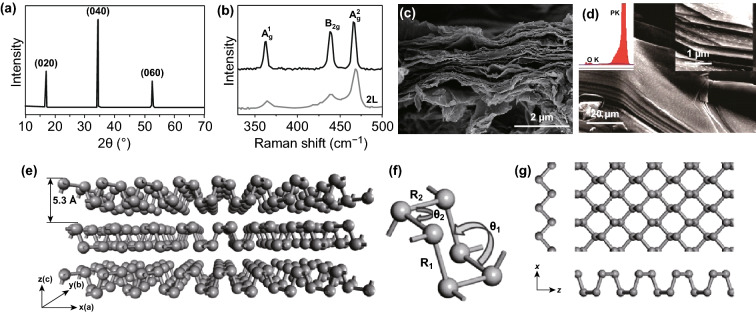
**a** XRD [77] and **b** Raman spectra [78] of BP. SEM image of the layered structure of **c** graphene and **d** BP. **e** Crystal structure and **f** detailed structure parameter (bond length and bond angle) of BP [79]. **g** The top view of BP crystal structure [78]. Adapted from Refs. [77–79] with permission

The crystal structure of BP was first determined in 1935 via the X-ray diffraction (XRD) [76], and it (Fig. 4a) reveals three peaks that are indexed to the (020), (040), and (060) planes in the 2θ range of 10°–70° [77]. And the Raman spectra of BP is shown in Fig. 4b, in which three typical Raman peaks correspond to A_g_^1^, B_2g_, and A_g_^2^ modes [78].

From the macroscopic view, scanning electron microscopy (SEM) image of graphene and BP as shown in Fig. 4c, d, it can be seen that BP has the layered wrinkled structure similar to graphene. The stacked layers are held together by weak van der Waals forces, which is why we can obtain phosphorene by top-down exfoliation method. In each puckered layer, the unit cell comprises two rows of parallel atomic layer in which consist of eight P atoms. And the unit cell is side-centered orthorhombic with lattice constants *a *= 4.47 Å and *b *= 3.34 Å.

From the microscopic perspective, as shown in Fig. 4e, we can see that every P atom is linked with three neighbor atoms [29]. Figure 4f shows the details of the BP crystal structure, the P–P distance (*R*_1_) between top and bottom atoms is 2.28 Å, and the corresponding angle (θ_1_) is 102.42°. The bond length (*R*_2_) with the nearest atoms is 2.25 Å, and the bond angle (θ_2_) is 96.16° [79]. The top view of BP microstructure is shown in Fig. 4g, the P atoms form zigzag (ZZ) and armchair (AZ) along x and z direction. This crystal structure is anisotropic, which endows BP with various physicochemical properties in different crystal orientation [80].

### Properties

#### Mechanical Properties

Anisotropic crystal structure endows BP with anisotropic mechanical properties, which can be illustrated by Young’s modulus. The Young’s modulus is an important index to measure the mechanical strength of materials. It is reported that the Young’s modulus of graphene, MoS_2_, and h-BN are 1.0 [81], 0.33 [82], and 0.25 [15] TPa, respectively. But the Young’s modulus of BP is relatively small, 0.166 TPa in ZZ direction and 0.044 TPa in AZ direction [83]. This smaller value may be caused by two aspects: (1) weak P–P bond strength and (2) the compromised dihedral angles rather than bond length stretch when tensile strain is applied, which gives BP a strong mechanical flexibility. This feature makes BP a good choice for practical large-magnitude-strain engineering (such as flexible electronics, ultra-light materials, etc.).

#### Electrical Properties

The most fascinating electrical property of BP is its tunable band gap. The band gap is one of the most important properties in electronic material, which ranges from zero (in metals) to several electron volts (in insulators) [17]. We have known that graphene is a kind of 2D material with zero band gap, so graphene is more like a metallic conductor [84], which is also the most important reason to limit the development of graphene. Whereas BP has a tunable band gap with thickness-dependent, in other words, the energy gap of BP increases with the number of layers decreasing. This makes BP a natural p-type semiconductor, while giving BP a good carrier mobility [85].

In fact, as early as 1935, Robert [86] determined that the energy gap of bulk BP was 0.3 eV, and its hole mobility and electron mobility at room temperature were 350 and 220 cm^2^ V^−1^ s^−1^, respectively. However, these electrical properties changed greatly when the 3D structure of BP changed to the 2D structure. Theoretical calculation predicted that the monolayer BP possesses direct energy gap of ~ 2.0 eV. The carrier mobility of few-layer BP can reach 1000 cm^2^ V^−1^ s^−1^ [37]. However, these electrical properties are often related to the quality of the phosphorene prepared. The energy gap of the monolayer BP prepared by Liu et al. [44] is 1.45 eV, slightly lower than the theoretical value. The mobility of the few-layer BP prepared by Erande et al. [64] was only 7.3 cm^2^ V^−1^ s^−1^, while that of the large-scale BP film prepared by Xu et al. [75] was up to 1200 cm^2^ V^−1^ s^−1^. Therefore, to make full use of the electrical properties of BP, the key lies in the preparation of high-quality phosphorene.

#### Electrochemical Properties

The interlayer distance of BP (5.3Å) is larger than that of graphene (3.3Å) [87, 88] which makes the intercalation of ions easier, thus provides better ionic conductivity. The first-principles calculations describe that the folded structure of BP can provides an ultrafast diffusion channel for Li^+^, Na^+^, and Mg^2+^ [89, 90]. Moreover, each P atom can bonding with three Li or Na form Li_3_P and Na_3_P, this provides BP high theoretical specific capacity of 2596 mAh g^−1^ which is seven times that of graphite (372 mAh g^−1^) [91]. And the working voltage range of BP (0.4–1.2 V) [92] in LIBs is also higher than that of graphite (0–0.25 V) [93]. In addition, the BP can effectively adsorb S atom and catalyze polysulfide conversion in LSBs [94]. On the other hand, BP is the allotrope with the most stable thermodynamic properties in phosphorus, with a good electric conductivity of ~ 300 S/m, which is much higher than allotrope RP (~ 10^−16^ S m^−1^) [95]. Based on these excellent electrochemical properties, BP has attracted considerable interest as a candidate material for energy storage devices.

## Environmental Instability and Passivation

### Environmental Instability

**Fig. 5:**
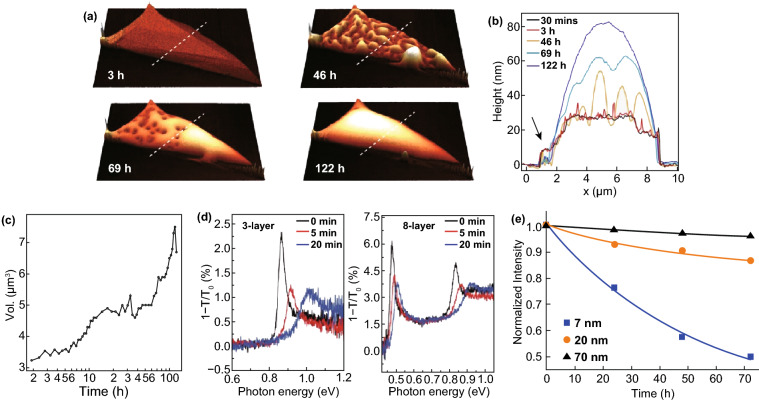
**a** AFM image, **b** height change curves and **c** volume change curves of BP in different exposed times [47]. **d** The blueshift of 3-layer and 8-layer BP in IR spectra [96]. **e** The evolution of the intensity of Raman normalized peak A_g_^1^ of BP with exposed time [97]. Adapted from Refs. [47, 96, 97] with permission

These excellent properties of BP come from its 2D structure. However, a large number of experiments have shown that prepared phosphorene is easy to decompose under the ambient conditions. Island et al. [47] used the atomic mechanics microscope (AFM) to continuously record the state of few-layer BP flake exposed to environmental condition for 5 days, and used the field effect transistor (FET) to measure the electrical property of BP during this period. The AFM image (Fig. 5a), height (Fig. 5b), and volume change curves (Fig. 5c) reveal that bubbles occur on the surface of BP flake due to water absorption with the increase in expose time. After 5 days, water completely covered the BP flake forming a convex meniscus with a height change of excess twice and a volume change of more than 200%. The FET devices show that the electrical properties of BP will decline within several hours to days due to water absorption. Wang et al. [96] also found the vulnerability of phosphorene exposure to air. It was found that the degradation of BP would cause blueshift of all resonance peaks in infrared (IR) absorption and PL spectra, especially in the thinner BP (Fig. 5d), which indicates that the structure of BP has been destroyed. Abellán et al. [97] further discussed the influence of thickness on BP degradation. By measuring the intensity of Raman normalized peak A_g_^1^ of BP (Fig. 5e), the result reflected that BP with thickness less than 10 nm showed faster degradation under ambient conditions, which indicated that the degradation of BP was also thickness-dependent. The structural failure caused by the degradation will affect the intrinsic properties of phosphorene and bring difficulties to its practical application in device [98, 99]. However, on the other hand, Wu et al. [100] suggested that the degradation of BP actually has a positive side. This is because they observed a significant friction reduction in the degraded area of BP nanoflakes, indicating that environmental degradation of BP significantly facilitates its lubrication behavior. Anyway, it is necessary to make clear the degradation mechanism of phosphorene.

Since each P atom in BP has one lone pair electrons, which makes BP easy to react with O_2_ to form P_x_O_y_, thus resulting in the environmental instability [101]. Some studies suggest that the degradation of BP is the result of the interaction of water, oxygen and light [102, 103]. Zhou et al. [104] proposed that the ambient degradation of BP includes three steps: superoxide formation under light, superoxide dissociation, and final breakdown under the action of water. In addition, some researches have investigated in detail the effect of H_2_O and O_2_ on BP decomposition. It was found that BP could be stably preserved in deoxygenated water owing to the hydrophobic of pristine BP, but once BP reacted with O_2_, it would turn into super-hydrophilic to promote further oxidation [105, 106]. Although there have been various theoretical and experimental studies on BP degradation mechanism in recent years, there is still a long way to go to fundamentally solve the problem of BP degradation. Therefore, in order to improve the long-term stability of BP and give full play to its potential in device application, it is necessary to develop passivation methods to protect phosphonene from degradation [107].

### Passivation

#### Coating

Coating a protective layer on phosphorene surface can effectively prevent phosphorene contact with the ambient conditions. There are various coatings, such as inorganic oxide [108–111], organics [112–114], polymer [115], and ionic liquid [97].

SiO_2_ shells are regard as a kind of excellent inorganic oxide coating due to its optically transparent which can maintain the optical properties of the core materials. Li et al. [109] used methyltrithoxysilane (MTEOS) to coat a hydrophobic SiO_2_ shell on the surface of BP nanosheets which could effectively prevent BP from contacting with water, thus mitigating the degradation. And then a second hydrophilic SiO_2_ shell was coated on the surface of hydrophobic BP@SiO_2_ nanocomposite by tetraethoxysilane (TEOS). The double SiO_2_ shell can not only maintain the intrinsic properties of BP, but also the hydrophilic surface can expand its application range. Liang et al. [114] coated hexamethylenediamine (HMA) organics on the surface of phosphorene. Compared with the complete decomposition of bare phosphorene within 12 h under the ambient environment, the phosphorene@HMA compound could be preserved stably in aqueous solution for more than one month.

**Fig. 6:**
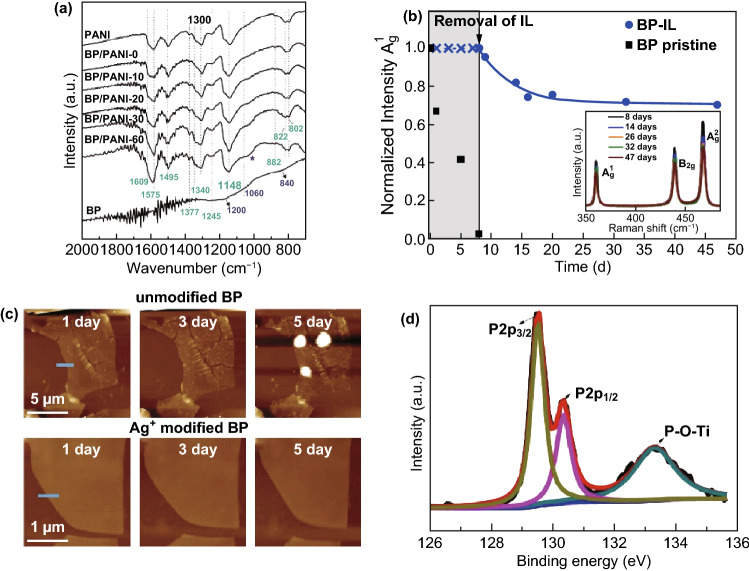
**a** FTIR spectra of BP@PANI under different exposed time [115]. **b** The evolution of Raman normalized peak A_g_^1^ of BP-IL and pristine BP with time [97]. **c** AFM image of unmodified BP and Ag^+^ modified BP under different air exposed time [49]. **d** XPS of BP/Ti_3_C_2_ heterostructure [117]. Adapted from Refs. [49, 97, 115, 117] with permission

Fonsaca et al. [115] reported a liquid/liquid interfacial method for covering BP sheets with the polymer polyaniline (PANI). The degradation degree of BP can be observed from the position and intensity of the Fourier Transform Infrared (FTIR) spectra (Fig. 6a). It was found that the BP in PANI polymer began to oxidize in about 20 days and completely degraded in 60 days under ambient conditions. However, the bare BP was oxidized within 3 days and completely decomposed after 15 days, which indicated the coating of PANI polymer could increase the life of BP by about 600%. Besides, Abellán et al. [97] proposed an effective ionic liquid passivation route with 1-butyl-3-methylimidazolium tetrafluoroborate (BMIM-BF4) as the coating of BP flake. The intensity curve of Raman normalized peak A_g_^1^ of BP (Fig. 6b) shows that BP flake can be stored in the BMIM-BF4 ionic liquid for 7 days without degradation. When the ionic liquid was removed, the intensity of Raman peak decrease about 30% after 47 days, which is mainly caused by the continuous laser exposure in the process of measuring Raman spectrum. The results suggested that the simple and effective BMIM-BF4 ionic liquid passivation method could effectively suppressed the degradation of BP.

#### Surface Modification

The lone electron pair in each P atom makes BP quite active in the air. Therefore, the surface modification methods are being explored to occupy the active lone electron pair of BP, thus improving its structural stability under environmental conditions.

Guo et al. [49] made use of the cation–π interactions to make Ag^+^ adsorbed spontaneously on the surface of BP sheets, thereby passivating the lone pair electrons of BP. As can be seen from AFM images in Fig. 6c, bubbles appeared on the surface of unmodified BP after 5 days of air exposure, while no bubbles and corrosion could be observed on the surface of Ag^+^ modified BP. Moreover, the modification of Ag^+^ also significantly improved the electrical properties of BP, greatly increasing the hole mobility from 796 to 1666 cm^2^ V^−1^ s^−1^. In addition, Fe^3+^, Mg^2+^, and Hg^2+^ can also be used as metal-ion modification methods for BP passivation. Zhao et al. [116] adopted TiL_4_ as the surface ligand of BP sheets, which can react with BP to form TiL_4_-coordinated BP. The optical absorbance intensity spectrum showed that the intensity attenuation of BP@TiL_4_ was only 8% after 1 week, while that of bare BP was 55% after 72 h.

Additionally, the surface modification of BP can also be realized by bonding other materials with BP to form heterostructure. Li et al. [117] proposed to combine BP with Ti_3_C_2_ to construct 2D BP/Ti_3_C_2_ heterostructure. It was found that compared with the naked BP, the BP/Ti_3_C_2_ appeared the red-shifts of Raman peaks, illustrating that new chemical bond was formed [118]. X-ray photoelectron spectroscopy (XPS) (Fig. 6d) further confirmed the formation of P-O-Ti bonds between BP and Ti_3_C_2_. Li et al. suggested that the formed chemical bonds can stabilize the structure of BP and have a certain improvement effect on its environmental stability.

#### Doping

Doping has been proven to be a simple and efficient method to adjust the intrinsic properties of 2D materials [119–121]. For BP, by introducing heteroatoms, the 2D structure can be tuned to improve its physical and chemical properties, including environmental instability. The types and ways of doping are varied. Hu et al. [122] divided the doping engineering into substitution doping, intercalation doping, surface charge transfer doping and electrostatic carrier doping.

Tang et al. [123] prepared large-scale fluorinated phosphorene (FP) by doping F into phosphorene via a facile one-step ionic liquid-assisted electrochemical exfoliation route. The FP inherits the antioxidation and antihydration properties of highly electronegative fluorine atoms, endowing it an air-stable photothermal properties that can be preserved for more than a week. Xu et al. [124] successfully induced the doping of N to few-layer BP by coating the BP surface with Si_x_N_y_ dielectric. The electron doping not only optimized the air stability, kept the electrical properties unchanged for more than one month, but also improved the electron mobility to ~ 176 cm^2^ V^−1^ s^−1^. Lv et al. [125] proposed S-doping can also improve the stability and electrical properties.

Through passivation means such as coating, surface modification and doping, the structural breakdown of BP caused by environmental exposure can be effectively avoided. The long-term stability of BP is the basis of its further application.

## Applications in Lithium-ion Batteries

Possessing basic environmental stability makes BP a strong candidate material in the field of energy storage. It has been reported that BP can be used as electrode material for Li^+^, Na^+^, K^+^, Mg^+^-ion batteries due to its high theoretical specific capacity and good electric conductivity [90, 95, 126, 127]. Among these electrochemical energy storage devices, LIBs have been widely applied in many fields with its most mature system. For this reason, in this section, we will discuss in detail the application and challenges of BP as the anode material for LIBs.

Since Sony commercialized lithium-ion batteries in 1990, LIBs had become one of the research hotspots in the field of new energy due to its high specific capacity, long cycle life, no pollution and good safety. LIBs are typically composed of an anode, a cathode and electrolyte, in which the charge flow is generated by the intercalation and deintercalation of Li^+^ between anode and cathode [128]. As we all know, the choice of electrode materials plays a crucial role in the performance of LIBs.

**Fig. 7:**
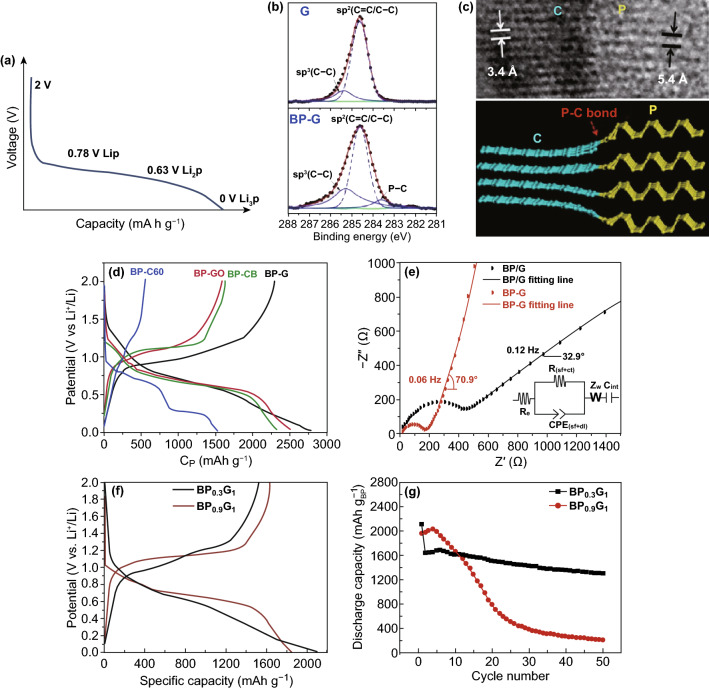
**a** The multistep lithiation process of phosphorene [127]. **b** XPS of G and BP-G composition, **c** A P–C bonding type in BP-G composition, **d** First charge/discharge curves of four anode materials (BP-C_60_, BPGO, BP-CB and BP-G) at 0.2C ratio, **e** EIS of BP-G composite and BP/G mixture [136]. **f** The first discharge–charge profiles and **g** cycle performance of BP_0.3_-G_1_ and BP_0.9_-G_1_ [138]. Adapted from Refs. [127, 136, 138] with permission

BP has a high theoretical capacity of 2596 mAh g^−1^, which provides a capacity basis for LIBs. Meanwhile, BP has good electric conductivity of ~ 300 S m^−1^, which makes it possible to prepare high-rate LIBs with fast charged and discharged. These properties show great potential in the fabrication of high-performance LIBs. Properties depend on structure. The unique wrinkled structure of BP provides more space for intercalation of Li^+^, in which Li atoms can combine with P atoms to form strong bond [34, 79, 129]. Qiu et al. [127] suggested that this lithiation process can be divided into BP → LiP → Li_2_P → Li_3_P as shown in Fig. 7a. This strong bond energy provides low diffusion energy barriers (0.08 eV) for the mobility of Li atoms [129, 130]. Zhang et al. [131] proposed that the diffusion mobility of Li^+^ in BP along the ZZ direction is about 10^7^–10^11^ times of that in the AC direction through theoretical calculation. This directional diffusion mobility is vastly superior than that of other 2D materials, such as graphene and MoS_2_, which renders BP possess the ultrafast charging/discharging characteristics. Furthermore, the 2D structure of BP shows high reversible during lithium intercalation. In other words, the volume change of monolayer BP is only 0.2% compared with a large volume change of 300% of bulk BP in the process of lithiation [132, 133]. Large storage space for Li^+^, ultrafast Li^+^ diffusion rate, and reversible stable structure make thin-layer BP has become one of the most promising candidate of anode materials of LIBs in the future [134]. Accordingly, some experiments have been carried out to use phosphorene as the anode material of LIBs.

Del Rio Castillo et al. [58] prepared few-layer BP with average lateral dimension up to 30 nm and the average thickness of 13 layers by liquid-phase exfoliation method as the anode of LIBs. Furthermore, the electrochemical properties of obtained few-layer BP was tested with half-cell, which showed an initial capacity of 1732 mAh g^−1^ at a current density of 100 mA g^−1^. However, the electrode appeared a large capacity fades within the first 10 cycles. After 100 charge/discharge cycles, the specific capacity decayed to ~ 480 mAh g^−1^. Zhang et al. [135] also prepared few-layer BP (5–12 layers) by liquid-phase exfoliation method. However, the few-layer BP electrode exhibited the same rapid capacity fade, resulting in the Coulombic efficiency (CE) of only 11.4% and reversible specific capacity of only 210 mAh g^−1^. The result is not satisfactory. They suggested that these poor performances might be due to the too thick layers resulting in the large volume variation during the cycle.

We have known that monolayer BP possesses the high structural reversible (only 0.2% volume change), as a result, it can be determined that reducing the number of BP layers can effectively solve the volume expansion problem of thick-layer BP [127]. However, the technology of preparation and passivation of thin-layer BP are not mature. So then, the direct use of phosphorene as anode material for LIBs needs further exploration. In view of this, in order to overcome the issues of fast capacity fading, low Coulombic efficiency and low reversible capacities caused by volume expansion of BP during lithiation/delithiation process, researchers proposed to construct BP-based composite by combining BP with other materials [35].

### BP/Carbon Composites

BP can easily combine with different carbon materials to form stable composites. Sun et al. [136] adopted four different carbon sources (graphite (G), graphite oxide (GO), carbon black (CB) and fullerene (C_60_)) to prepare BP/C composites by a simple and efficient high energy mechanical milling (HEMM) method, and studied the effect of four different phosphorus–carbon (P–C) bonds on the electrochemical properties. The result indicates that graphite can supply *sp*^2^-bonded C atoms (Fig. 7b), which can bond with P atoms at the edge of BP to form the shortest and the most stable *sp*^2^ P–C bond in aromatic ring (Fig. 7c). In addition, the BP-G composites show the highest percentage concentration of P–C bond compared with the other three composites, which endows BP-G the highest initial discharge capacity of 2786 mAh g^−1^ (Fig. 7d) at the current destiny of 0.2 C, 80% capacity retention after 100 cycles and high-rate capability. To investigate in detail the role of the P–C bond in improving electrochemical performance. Sun et al. additionally prepared the BP/G mixture (without P–C bonds) to compare with BP-G composite. After 10 stable cycles, the electrochemical impedance spectroscopy (EIS) of the two electrodes materials was measured, and the results are shown in Fig. 7e. Since the single semicircle is observed in the EIS, the impedance can be attributed to the combination of the surface film resistance (*R*_sf_) and the charge transfer resistance (*R*_ct_) (at the interface between the electrode and electrolyte), which is called *R*_(sf+ct)_. And the *R*_(sf+ct)_ fitting parameter of BP-G (177.4 Ω) is much lower than that of BP/G (484.2 Ω), which means that the P–C bonds in BP-G composite can not only form a stable surface film on the electrode surface, but also provide a faster charge-transfer channel at the interface between the electrode and the electrolyte. These stable structures provided by P–C bond greatly reduces the volume expansion of BP during lithiation/delithiation process. This is why combining BP with carbon materials can significantly improve its electrochemical performance. In addition, Ramireddy et al. [137] also synthetized the BP-G composites by HEMM method. The BP-G exhibite initial discharge capacity of 1930 mAh/gand CE of 86.9% in the 2.0–0.01 V wide potential window and current of 100 mA g^−1^. However, under this wide potential window, it can be observed that the BP-G electrode gradually disintegrate and delaminate from collectors, resulting in the capacity decaying at a faster rate. But, when the potential windows was restricted to 2.0–0.67 V, a relatively stable capacity of ~ 700 mAh g^−1^ can be maintained. This indicates that the P–C bond is easier to play a role within the appropriate voltage range.

In order to give full play to the improvement effect of P–C bond on the electrochemical performance of BP. Some researchers began to investigate the effects of different BP/G molar ratios on the electrochemical properties of BP-G composites. Recently, Shin et al. [138] prepared BP_x_-G_y_ composites with two different molar ratios (x:y) via HEMM method for LIBs anode. Firstly, BP_0.9_-G_1_ composite was synthetized at different milling times, and the initial discharge capacity of BP_0.9_-G_1_ prepared by 6 h-milling (1847 mAh g^−1^) is higher than that of 1 h-milling (1126 mAh g^−1^) at the current density of 200 mA g^−1^. This means that the formation of P–C chemical bond in BP-G composite requires a certain milling time in the process of HEMM. Secondly, BP_0.3_-G_1_ composite with low molar ratio was synthetized and compared with BP_0.9_-G_1_. As can be observed from Fig. 7f, both of these two electrodes have high first discharge and charge capacity, but BP_0.9_-G_1_ shows relatively long discharge (lithiation process) and charge (delithiation process) voltage plateau. Moreover, BP_0.3_-G_1_ shows two charge voltage plateaus at about 1.0 and 1.2 V, which might be due to the different molar ratios leading to the various lithiation and delithiation process. As shown in Fig. 7g, the low molar ratio anode material (BP_0.3_-G_1_) possesses relatively stable cycle performance compared with the rapid capacity fade of BP_0.9_-G_1_, which reveals that the molar ratio of BP/G affects the crystal structural integrity of BP-G composite.

**Fig. 8:**
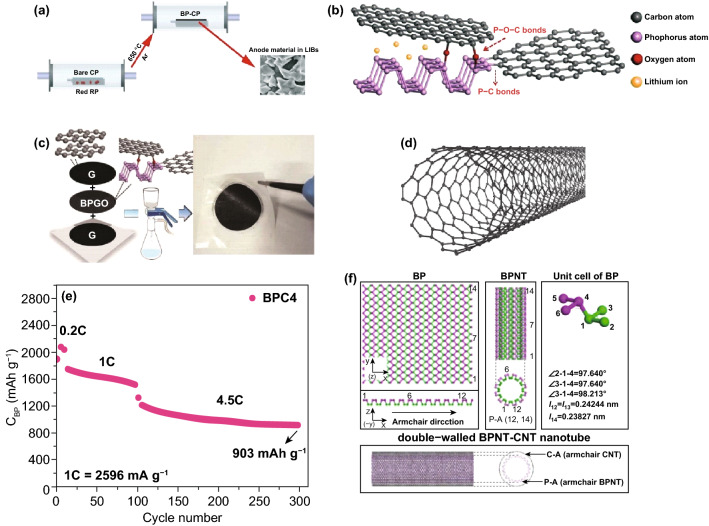
**a** The thermal-vaporization deposition process of BP-CP synthesis [73]. **b** The chemical bonds of BPGO and **c** G-BPGO-G thin film preparation process [139]. **d** Atomic structure of CNT. **e** High-rate cycle stability of BP-CNT at 1C and 4.5C [141]. **f** The theoretical model of BPNT-CNT double-walled nanotube structure [142]. Adapted from Refs. [73, 139, 141, 142] with permission

In addition to HEMM method, several other methods of compounding BP with carbon materials have been reported. Jiang et al. [73] used the efficient thermal-vaporization deposition approach (Fig. 8a) to convert RP into BP and make it grow directly on the surface of carbon paper (CP). The BP–CP composites exhibited high initial discharge capacity (2168 mAh g^−1^ at 0.1C) and excellent cycling stability (75.58% capacity retention after 200 cycles) as the anode of LIBs. Liu et al. [139] firstly synthesized BP-GO composites by solvothermal method at 140 °C, and then constructed the G-BPGO-G thin film with sandwich structure by a facile vacuum filtration approach. In the solvothermal process, the P–C and P-O-C bonds were formed between BP and GO (Fig. 8b). During the vacuum filtration process, as shown in Fig. 8c, the active BPGO was coated by graphene layers to form the sandwich structure. Stable chemical bonds and the sandwich structure can not only protect BP from rapid volume expansion in the process of lithiation and delithiation, but also the two graphene stacks can protect BP from oxidation and can replace copper foil as current collector of LIBs. These characteristics make G-BPGO-G possesses reversible capacities of 1401 mAh g^−1^ at current density of 100 mA g^−1^ in the 200th cycle.

Furthermore, there is a special structure of carbon materials–carbon nanotubes (CNT) (Fig. 8d), which are mainly composed of *sp*^2^ hybrid carbon atoms arranged into hexagon to form several to dozens of coaxial tubes [140]. Shishavan et al. [141] prepared a new BP-CNT composite by surface oxidation-assisted chemical bonding procedure for the first time. After a long time of ball-milling and appropriate air exposure, the oxide layer appeared on the surface of BP as well as the carboxyl and hydroxyl functional groups appeared on multiwall CNT. And then, through the cross-linking effect of binder, two types of strong connections occur between these raw materials: the formation of P-O-C bonds and dehydration reaction. Finally, the discharge capacity of the BP-CNT electrode maintained at 1681 mAh g^−1^ (at 0.2C) with 87.5% capacity retention after 400 cycles as well as showed a high-rate cycle stability at 1C and 4.5C (as shown in Fig. 8e). In addition, there are reports indicated that it is possible to restructure BP into CNT-like nanotubes (BPNT) through theoretical calculation [142, 143]. Shi et al. [142] proposed that this concept could be used to construct the double-walled BPNT-CNT nanotube composites (Fig. 8f) via theoretical model analysis, which provides a new idea and theoretical basis for the establishment of BP-based composite with special structure.

These theoretical and experimental researches show that the P-C bonds can exert its stability by selecting the appropriate carbon source, BP/G molar ratio, preparation method and potential window. This stable chemical bond can not only solve the problem of volume expansion in the charging and discharging process, but also provide stable surface film and faster charge-transfer channel at the interface, thereby optimizing the electric conductivity, reversible specific capacity and Coulombic efficiency of BP anode material [136, 138, 142]. In addition, some noncarbon materials can also form stable composites with BP.

### BP/Noncarbon Composites

**Fig. 9:**
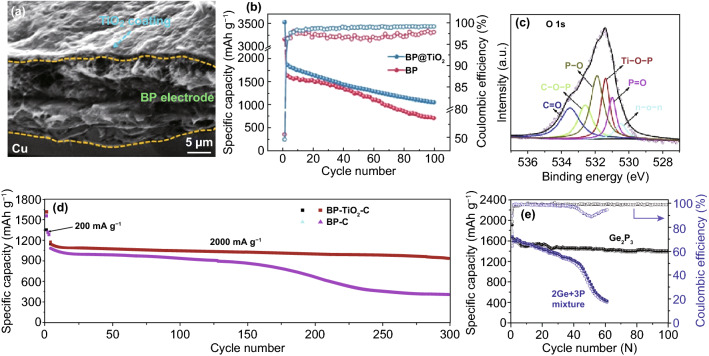
**a** SEM and **b** cycling performance of BP@TiO_2_ composite [146]. **c** XPS spectra of O 1 s of BP-TiO_2_-C composite, **d** the cycle performance of BP-TiO_2_-C and BP-C composite [147]. **e** The cycle stability of Ge_2_P_3_ and 2Ge + 3P electrode [153]. Adapted from Refs. [146, 147, 153] with permission

TiO_2_ has zero-strain characteristic (volume change < 4%) and can provide the rapid Li^+^ migration rate due to the formation of Li_x_TiO_2_ in the process of lithiation, which is considered as an electrode material with good crystal structure stability [144, 145]. From this point of view, combining TiO_2_ with BP can effectively improve the crystal stability and ionic conductivity of BP. Luo et al. [146] fabricated BP@TiO_2_ composites by coating about 50 nm-thick TiO_2_ layer on the BP particles surface (Fig. 9a) in an electron-beam evaporation system. The ultrathin TiO_2_ layer can prevent BP from contacting with electrolyte, thus reducing the corrosion effect of the electrolyte. On the other hand, the Li_x_Ti_y_O_z_ layer formed on BP surface during the lithiation process can restrain the further generation of the solid electrolyte interface (SEI) film on the surface of BP. Finally, the specific capacity, coulomb efficiency and cycle stability (as shown in Fig. 9b) of BP are improved. In addition, Zhou et al. [147] reported a BP-TiO_2_-C nanocomposite synthesized via HEMM method. The XPS spectra in Fig. 9c reveals that there is Ti–O-P bonds (the peak ~ 531.3 eV) in the BP-TiO_2_-C, which may be caused by the combination of TiO_2_ and hydroxyl (formed on BP surface during ball-milling). Moreover, some other bonds were also formed, such as P–C, P-O, C-O, and C-O-P. On the other hand, it is further confirmed that TiO_2_ can be transformed into Li_x_TiO_2_ during the initial discharge process. The formed strong Ti–O-P bonds and Li_x_TiO_2_ not only can keep the stability of internal active materials but also create conditions for the transfer of interfacial electron, which endows the BP-TiO_2_-C electrode high capacity of ~ 935.8 mAh g^−1^ after 300 cycles (85.3% capacity retention) even at high current density of 2000 mA g^−1^, which is much higher than the BP-C electrode without TiO_2_ (Fig. 9d). The results show that Ti–O-P bond can maintain the electrochemical performance of BP better than P–C bond.

Metal–organic frameworks (MOFs) is a kind of crystalline porous materials with chemical and structural stability, which is based on a type of network structure formed via self-assembly between metal and organic coordination bond [148, 149]. Considering that MOFs can solve the problem of volume change, Jin et al. [150] fabricated few-layer BP-NiCo MOF hybrid through directly anchored NiCo MOF on the few-layer BP using a facile solution reaction route in a mixed solution of Ni^2+^, Co^2+^ and benzenedicarboxylic acid (BDC). The carboxylate groups in BDC^2−^ can not only chelate with Ni^2+^ and Co^2+^ but also bonding with BP, thus obtaining a stable hybrid structure. The BP-NiCo MOF with 2D and porous nanostructure can provide high specific surface area for charge transport as well as can buffer the volume change during the cycle, which leading to a good reversible capacity (853 mAh g^−1^ at 500 mA g^−1^ after 200 cycles) and high-rate cycle performance (398 mAh g^−1^ at 5000 mA g^−1^ after 1000 cycles).

In the 2D material family, some other 2D materials, such as h-BN [151], MXenes [152], and 2D metal phosphides (GeP [153], Cu_2_P_7_ [154], etc.), are easy to form stable hybrid with BP due to their similar 2D structure. Through theoretical calculation, Chowdhury et al. [151] proposed that h-BN could be used as an effective capping agent for BP. It is found that the BP-(h-BN) composite has insignificant volume changes (~ 1.5–2.0%) and can steadily abosorb up to 20 Li atoms, therefore possesses a theoretical capacity of 607 mAh g^−1^. GeP is an emerging and typical 2D semiconductor material with strong anisotropic physical properties [155]. Li et al. [153] successfully synthesized Ge_2_P_3_ composites via facile HEMM method, which derived from the mixture of layered BP and GeP. The Ge_2_P_3_ electrode delivered a fairish initial discharge (1795 mAh g^−1^) and charge (1610 mAh g^−1^) specific capacity, which endows an initial CE of 89%. Moreover, Fig. 9e indicates that the Ge_2_P_3_ electrode possesses excellent cycle stability (100 cycles maintain at 1380 mAh g^−1^), which is better than the electrode of 2Ge + 3P mixture (60 cycles maintain at 410 mAh g^−1^).

BP can combine with different materials to form stable BP-based composites through various methods. The chemical bond formed in the composite process can not only stabilize 2D crystal structure of BP to avoid large volume change during the cycle, but also improve the charge conductivity, thus optimizing the performance of LIBs. Table 2 summarizes the different BP-based composites as well as their performance as the anode of LIBs. These BP-based composites have shown great potential in improving the performance of LIBs.

**Table 2:** Summary of BP-based composites as the anode of LIBs

BP-based composites		Experiment	Initial discharge capacity	Cycle performance	References
BP/Carbon composites	BP-CP	BP directly grown on the carbon paper by thermal-vaporization deposition method	2168 mAh g^−1^ at 0.1C	1677 mAh g^−1^ at 0.1C after 200 cycles	[73]
	BP-G	Compositing BP and graphite by HEMM	2786 mAh g^−1^ at 0.2C	1849 mAh g^−1^ at 0.2C after 100 cycles	[136]
	BP_0.3_-G_1_	Compositing 43.6 wt.% of BP and 56.4 wt.% of graphite by HEMM	1600 mAh g^−1^ at 200 mA g^−1^	1300 mAh g^−1^ at 200 mA g^−1^ after 50 cycles	[138]
	G-BPGO-G	Constructing sandwich structural G-BPGO-G by solvothermal reaction and vacuum filtration	1633 mAh g^−1^ at 100 mA g^−1^	1401 mAh g^−1^ at 100 mA g^−1^ after 200 cycles	[139]
	BP–CNT	Compositing BP and carbon nanotubes via a surface oxidation-assisted chemical bonding procedure	2073 mAh g^−1^ at 0.2C	1681 mAh g^−1^ at 0.2C after 400 cycles	[141]
BP/Non-Carbon composites	BP-TiO_2_-C	Compositing BP, TiO_2_ and graphite by HEMM	1581.1 mAh g^−1^ at 100 mA g^−1^	935.8 mAh g^−1^ at 2000 mA g^−1^ after 300 cycles	[147]
	BP-NiCo MOF	Through directly anchored NiCo MOF on the few-layer BP using a facile solution reaction route	2483 mAh g^−1^ at 100 mA g^−1^	853 mAh g^−1^ at 500 mA g^−1^ after 200 cycles	[150]
	Ge_2_P_3_	Compositing BP and GeP by HEMM	1795 mAh g^−1^ at 100 mA g^−1^	1380 mAh g^−1^ at 100 mA g^−1^ after 100 cycles	[153]

## Perspective and Conclusion

Due to its unique 2D structure, BP is endowed with a series of excellent properties, such as tunable bandgap structures, outstanding electrochemical properties, anisotropic mechanical, thermodynamic, and photoelectric properties. These excellent properties provide opportunities for the application of BP in photonics, electronics, sensors, ultra-light materials, energy storage devices, flexible electronics, and other fields. However, opportunities always coexist with challenges.

First of all, it is difficult to prepare 2D structural phosphorene. To stimulate the maximum potential of BP, we need to prepare large-scale uniform high-quality phosphorene. Therefore, we must improve the preparation methods, especially the bottom-up direct chemical synthesis method. Secondly, the environmental stability of 2D BP is poor, which brings great difficulties to the practical application of BP. So, it is urgent to make clear the degradation mechanism of BP and develop passivation methods to improve its long-term stability. Finally, although BP is a promising energy storage material, there are also some challenges in practical application. During the cycling of LIBs, due to the volume expansion of BP, it will cause problems such as fast capacity fading, low Coulombic efficiency and low reversible capacities. In order to solve these problems, we must continue to explore improved methods, such as BP composite with other materials. These challenges cannot be underestimated. To achieve the wide application of BP, we need to conduct a lot of research.

In this review, the different preparation methods of phosphorene are summarized and compared in detail. Although the “top-down method” is simple and low-cost, the size of the prepared phosphorene is not uniform and the yield is relatively low. The “bottom-up method” has great potential in the preparation of controllable large-scale and high-quality phosphorene, but it requires higher equipment conditions. In addition, we discuss the crystal structure and fundamental properties of BP. The environmental instability of BP poses a challenge to its practical application. But this problem can be significantly improved by means of coating, surface modification and doping, which lays a foundation for the further practical application of BP. Finally, the latest research results of BP-based anode materials in LIBs field are summarized and analyzed. However, due to the large volume variation of BP in the charging and discharging process, the performance of LIBs is seriously affected. A large number of experiments have shown that combining BP with other materials to construct BP-based composites can effectively improve this problem. This BP-based composites have shown great research value as the anode materials of LIBs.

In a word, the 2D structure endows BP with a variety of outstanding properties, which is considered as a promising new energy storage material. However, we know very little about BP. Hence, extensive theoretical and experimental researches are still needed to explore this potential advanced 2D material.
